# Phrygian cap deformity in a pediatric patient: a case report

**DOI:** 10.1093/jscr/rjag503

**Published:** 2026-06-26

**Authors:** Johann Paulo Guzman, Nour Yanna Atassi, Gazi Baderkhan Zibari, Hosein Mohammad Shokouh-Amiri, Kevin Neal Boykin

**Affiliations:** Department of Surgery, Willis Knighton Health, 2600 Greenwood Road, Shreveport, LA 71103, United States; Department of Surgery, Willis Knighton Health, 2600 Greenwood Road, Shreveport, LA 71103, United States; Department of Surgery, Willis Knighton Health, 2600 Greenwood Road, Shreveport, LA 71103, United States; Department of Surgery, Willis Knighton Health, 2600 Greenwood Road, Shreveport, LA 71103, United States; Department of Surgery, Willis Knighton Health, 2600 Greenwood Road, Shreveport, LA 71103, United States

**Keywords:** phrygian cap, cholecystectomy, gallbladder abnormality, choledochal cysts

## Abstract

The overall prevalence of congenital causes of gallstones is not well established. The Phrygian cap is a congenital anomaly of the gallbladder with an incidence of 4%. The literature provides a few case reports on Phrygian cap deformity with or without associated gallstones. This report presents a 15-year-old Hispanic, overweight female patient transferred to our service due to an aborted laparoscopic cholecystectomy. During the initial surgery, there was a suspicion of choledochal/gallbladder cyst. Due to intraoperative and technical difficulties, the patient was referred to our pediatric surgical service. All her prior imaging was unremarkable. A magnetic resonance cholangiopancreatogram revealed mild prominence of the cystic duct with a slightly ovoid in configuration, normal sized intrahepatic and common bile ducts, suspecting a type VI choledochal cyst. Endoscopic retrograde cholangiopancreatography, showed a phrygian cap deformity. The decision was made to proceed with a standard laparoscopic cholecystectomy. This report highlights that while cholecystectomy is curative, additional imaging may be needed to establish anatomy and diagnosis in unusual gallbladder anatomy.

## Introduction

Gallstone disease in children is evolving and can be attributed to multiple conditions. These conditions include the following: hemolytic disease, congenital, genetic, dietary, systemic, medication use, short gut syndrome and biliary motility problems that predisposes to stone development [1]. While hemolytic disease is a common comorbidity in adolescents with cholelithiasis, non-hemolytic risk factors have become increasingly significant contributors to its incidence. These factors include pregnancy, oral contraceptive use, and obesity [2–4]. Congenital anomalies of the gallbladder, such as double gallbladder, septate gallbladder, and gallbladder diverticulum, can be risk factors for bile stasis, inflammation, and stone formation as they alter bile flow. A large number of asymptomatic gallstones may never cause clinical problems [5, 6]. In children and infants, spontaneous resolution has been reported in ~16% and 34% of cases, respectively. When gallstones do become symptomatic, they most commonly present with pain in the right upper quadrant (85%–94% of cases), and less frequently with epigastric pain (~34%) [1, 7, 8].

The overall prevalence of congenital causes of gallstones is not well established in literature due to rarity, underdiagnosis (asymptomatic) and overlapping risk factors. Our literature review revealed a few case reports on Phrygian cap deformity with or without associated gallstones. Most case reports discuss patients of variable age range, from prenatal patients to adults. However, each had similar presentations, including confusion in diagnosis or an incidental finding of unrelated pathology [9–16]. This case discusses a pediatric patient presenting with an intra-operative dilemma during a routine laparoscopic cholecystectomy for cholelithiasis. The initial cholecystectomy was aborted and after extensive post-operative imaging and repeat laparoscopy a diagnosis of Phrygian cap deformity was established.

## Case report

**Figure 1:**
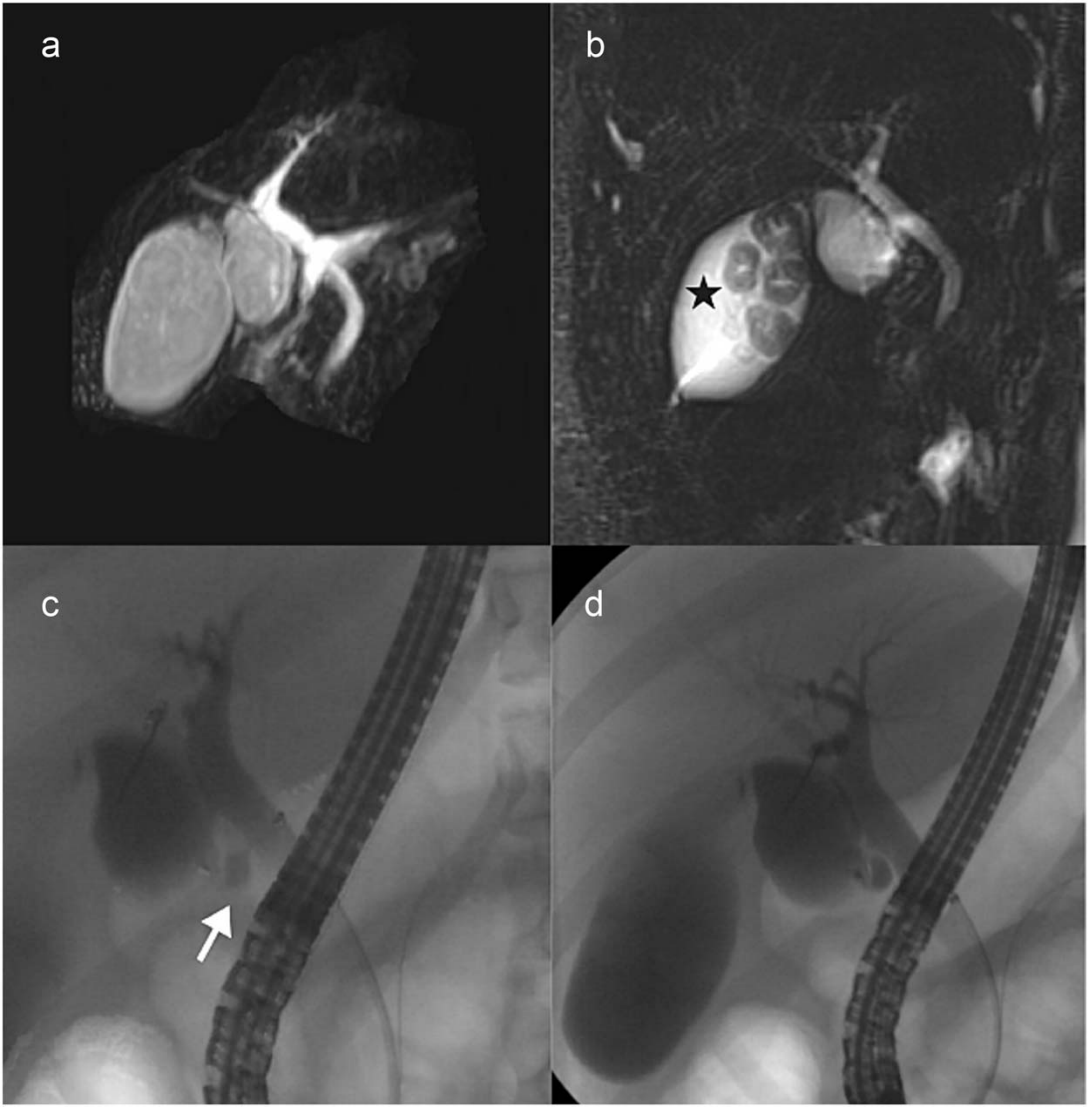
(a, b) Magnetic resonance cholangiopancreatogram showing cystic dilation of the cystic duct or a Phrygian cap deformity with gallstones (in asterisk). (c, d) Endoscopic retrograde cholangiopancreatogram showing the cystic dilation but normal sized cystic duct as it connects to the common bile duct (in arrow).

Informed consent from the parents and verbal assent from the patient were secured in accordance with the Willis Knighton Health Research Department’s policy. A 15-year-old overweight (BMI-28.4 kg/m^2^), Hispanic female patient was referred to the pediatric surgical service due to an initial suspicion of choledochal/gallbladder cyst. This was initially discovered during an attempted laparoscopic cholecystectomy for symptomatic cholelithiasis 5 months prior. Index laparoscopy revealed a scarred gallbladder with a proximal cystic structure. The initial surgery was aborted owing to concerns over a choledochal duct abnormality. No intra-operative cholangiogram was done at that time. The patient was discharged and in the interim, she continued to demonstrate occasional post-prandial mild right upper quadrant pain. Other review of systems revealed no history of jaundice, fever, acholic stools or bowel movement changes. The patient’s medical, surgical, family, personal and social history was unremarkable. A focused physical examination showed a flat, non-distended abdomen with healed trocar incision sites, and no masses on palpation. The initial ultrasound showed normal sized gallbladder, non-dilated bile ducts, and non-thickened walls associated with gallstones. Laboratories including bilirubin, alkaline phosphatase and liver function tests were within normal limits. Magnetic resonance cholangiopancreatogram (MRCP) showed mild prominence of the cystic duct, slightly ovoid in configuration measuring 2.3 × 1.9 × 2.2 cm. Additionally, it revealed normal sized intrahepatic and common bile ducts suggesting a possible type VI choledochal cyst. Endoscopic retrograde cholangiopancreatography (ERCP) was performed to delineate the anatomy and for operative planning (Fig. 1).

**Figure 2:**
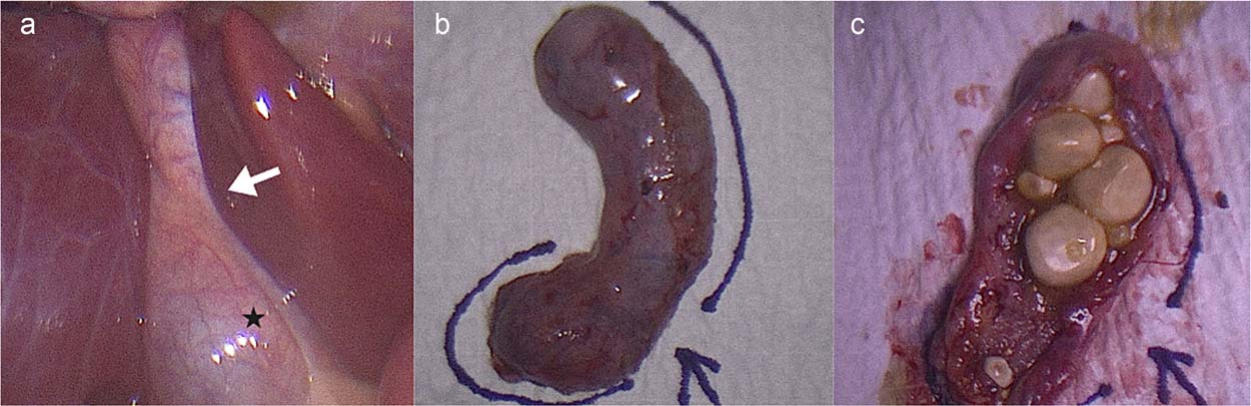
(a) Gallbladder with a hourglass shaped configuration (in arrow) causing the lower portion to look like a cystic structure (in asterisk). (b, c) Gallbladder straightened in the back table, no external deformity and no septation intraluminally (arrows drawn to orient where the area of in-folding was intraoperatively).

The decision was made to take the patient back to the operating room for a 4-port laparoscopic cholecystectomy. Intra-operatively, the upper portion of the gallbladder body was folded over the width of the gallbladder causing it to have a bilobed shape. This was made more prominent during gentle traction on the fundus to expose the cystic plate. Due to the bent shape, the lower portion of the gallbladder had the appearance of a cystic mass (Fig. 2). After achieving the critical view of safety, the cystic duct and cystic artery was ligated sequentially. The gallbladder was dissected off from the fossa and inspected in the back-table. Inspection of the gallbladder specimen revealed cholesterol stones, in addition to no septa.

Post-operatively, the patient developed a low grade fever due to atelectasis, however, this had resolved by the next day. The patient was discharged on the second post-operative day. Pathology reported multiple multifaceted tan-gray calculi within the lumen consistent with mild cholecystitis and cholelithiasis.

## Discussion

Initially described by Boyden, the deformity resembles a soft conical cap with the apex bent over worn by ancient Phrygians and was a symbol of liberty during the American Revolution [16]. This malformation of the gallbladder alone has no clinical manifestations; however, it may cause cholelithiasis, which leads us to this diagnosis. Although our extensive preoperative imaging workup did not significantly alter the surgical approach, congenital gallbladder anomalies should remain in the differential diagnosis. This is especially true when unusual anatomy is encountered during routine cholecystectomy. Admittedly, the anatomy may have been clarified at the index operation had an intraoperative cholangiogram or ultrasound been performed. This report proposes a diagnostic workup algorithm for the evaluation of suspected congenital gallbladder anomalies.

Most reports published that the incidence of a Phrygian cap is around 4%, but this could be grossly underestimated [7, 10–13]. In most cases with this deformity, there is a mucosal fold created which partially subdivides the lumen of the gallbladder, causing potential stasis and stone formation. A gallbladder septum is a differential in this situation that can be diagnosed with ultrasound, MRCP and ERCP and later can be confirmed by opening the specimen [1, 3]. Embryologically, during the fourth week of gestation, the liver, gallbladder and biliary tree arise as a ventral bud from the most caudal part of the foregut. The hepatic diverticulum differentiates caudally into the gallbladder. It is theorized a folding of the fundus during this phase of development leads to this deformity [9]. Other theories state that it could be due to excessive longitudinal growth of the gallbladder, leading to an eventual fold as it reaches the anterior border of the liver [14].

Phrygian cap deformities can be symptomatic with varying degrees of clinical presentation. This case adds to the anatomic variations of the gallbladder that the surgeon should be cognizant when managing gallbladder diseases. Appropriate work-up may be warranted to establish an accurate diagnosis and guide planning and management.
